# Gene expression profiling in pure neural leprosy: insights into pathogenesis and diagnostic biomarkers

**DOI:** 10.3389/fimmu.2025.1550687

**Published:** 2025-05-12

**Authors:** Mariana Martins de Athaide, Thyago Leal-Calvo, Tatiana Pereira Da Silva, Thabatta Leal Silveira Andrezo Rosa, Helen Ferreira, Bernardo Miguel de Oliveira Pascarelli, Ana Caroline Siquara de Sousa, Marcia Rodrigues Jardim, Roberta Olmo Pinheiro

**Affiliations:** ^1^ Leprosy Laboratory, Oswaldo Cruz Institute, Oswaldo Cruz Foundation, Rio de Janeiro, Brazil; ^2^ Innovative Genomics Institute, University of California, Berkeley, Berkeley, CA, United States; ^3^ Cellular Microbiology Laboratory, Oswaldo Cruz Foundation, Rio de Janeiro, Brazil; ^4^ Post-graduate Program in Neurology, Federal University of the State of Rio de Janeiro, Rio de Janeiro, Brazil; ^5^ Department of Neurology, Pedro Ernesto University Hospital, Rio de Janeiro State University, Rio de Janeiro, Brazil; ^6^ Rio de Janeiro Research Network on Neuroinflammation, Oswaldo Cruz Institute, Oswaldo Cruz Foundation, Rio de Janeiro, Brazil; ^7^ National Institute of Science and Technology on Neuroimmunomodulation, Oswaldo Cruz Institute, Oswaldo Cruz Foundation, Rio de Janeiro, Brazil

**Keywords:** pure neural leprosy, transcriptomic analysis, inflammasomes, autophagy, immunopathogenesis

## Abstract

**Introduction:**

Leprosy may affect skin and nerves, leading to permanent disabilities and deformities. Pure neural leprosy (PNL) lacks skin lesions, complicating diagnosis. Moreover there is no a specific treatment to control neural damage. Transcriptomic profiling may reveals unique gene expression changes in PNL nerves, shedding light on immune response and pathogenesis. These findings may guide early diagnosis and improve patient outcome.

**Methods:**

In the present study, we investigated the gene profiling of nerve samples from patients with PNL and revealed significant transcriptomic alterations compared to non-leprosy controls.

**Results:**

Principal Component Analysis (PCA) of the 500 most differentially expressed genes separated the groups, with 1,199 genes showing differential expression (|log_2_FC| ≥ 1, FDR ≤ 0.1). Downregulated genes included *GAS2L2*, *TRIM67*, *IL1RAPL1*, *MAP1LC3B2*, and *NTNG1*, implicated in neuronal development and autophagy, while upregulated genes were linked to immune responses. Functional analyses highlighted inflammasome activation and autophagy impairment in PNL, correlating with nerve inflammation and architecture loss.

**Discussion:**

We hope that our data will aid in identifying new markers, fostering strategies for early diagnosis, preventing disabilities, and improving the management of PNL patients.

## 1 Introduction

Despite advances in the treatment of leprosy, a long-time known infectious disease caused by *Mycobacterium leprae* and *M. lepromatosis* (1), it still remains prevalent worldwide, mainly in countries such as India, Brazil and Nepal, where its endemic status is reinforced by the high numbers of new cases each year (2). Leprosy is usually known for its skin lesions, but neural abnormalities are another hallmark of the disease and the basis of leprosy-associated disability (3, 4).

Nerve damage in leprosy can be induced directly by *M. leprae*, due to the uniqueness of this bacterium in having the ability to invade the Schwann cells of the peripheral nervous system (PNS) (5). However, immune-mediated mechanisms characteristic of the parasite-host interaction add severity to the impairment of neural function in symptomatic periods of the disease or in a silent way (6–8).

Pure neural leprosy (PNL) constitutes approximately 10% of all types of leprosy (9). However, the diagnosis of PNL remains a public health problem mainly due to the presence of nervous involvement, with or sensory impairment, in the absence of any sign of skin lesions (10, 11). An early diagnosis is crucial, as a delay in diagnosis and treatment can lead to serious disability.

Nerve biopsy is a common diagnostic method that may establish a diagnosis in many peripheral neuropathies of unexplained etiology (12). In leprosy, the demonstration of bacilli is not always possible on nerve samples, and, for the most part, histological findings are nonspecific (11). Although the classic forms of leprosy with skin lesions have well-described clinical characteristics in the literature, these aspects in PNL are still poorly studied. Therefore, the gene profile identification in PNL patients becomes an excellent strategy for understanding the mechanisms involved in the immune response and pathogenesis in this group compared to other non-leprosy neuropathies (13).

In the present study, we used host transcriptomic profiling by RNA-seq to explore differences in genes and pathways in leprosy and non-leprosy nerve samples. It is our hope that the data obtained contributes to the identification of new markers to the development of strategies and interventions for early diagnosis, helping to prevent disabilities aiming to impact the management of PNL patients.

## 2 Materials and methods

### 2.1 Study population and human nerve biopsy

The present investigation is a retrospective study of nerve biopsy samples collected from leprosy and control groups. Patients enrolled in this study were assisted at the Souza Araujo Outpatient Unit (Fundação Oswaldo Cruz, Rio de Janeiro, Brazil), a reference center for leprosy diagnosis and treatment, and all underwent a standardized clinical evaluation for leprosy. A total of 16 adult patients were included and the study population was divided into two groups: 1) patients diagnosed with pure neural leprosy (PNL) (n=7); and 2) non-leprosy neuropathy group (vascular neuropathy) (n=9). The patients had been previously diagnosed as PNL, in agreement with the criteria adopted in previous publications (10).

All patients underwent a complete clinical, electrophysiological, and laboratory evaluation before nerve biopsy was considered, ensuring exclusion of alternative diagnoses. Nerve biopsy was performed only in individuals whose neurological and electrophysiological examinations confirmed the presence of peripheral neuropathy, with leprosy as a suspected cause. After completion of the histopathological analysis, patients who presented signs of vasculitis were referred to the Neurology and Rheumatology Service of the Pedro Ernesto University Hospital (Rio de Janeiro State University - UERJ) for further evaluation of the etiology of vasculitis. Only patients with a confirmed diagnosis of isolated vasculitis of the peripheral nervous system (single organ vasculitis), a condition with no defined etiology and no known association with infectious, inflammatory, or neoplastic agents, were selected as controls in this study. As such, it is classified as a primary and localized vasculitis.

Of the 7 PNL patients, 4 were male and 3 were female. The mean age in this group was 56.7 (36–72 [min-max]). The non-leprosy neuropathy group counted 9 patients of which, 1 male and 8 females (**Table 1**). Samples from patients with vasculitis were used as a control for non-infectious inflammatory peripheral neuropathy and the mean age in this group was 64.8 (42–83 [min-max]). In this scenario, what distinguishes them is precisely the gene modulation induced by the presence of the bacillus.

**Table 1:** Clinical data from patients included in the present study.

Clinical Form	Sex	Age (years)	Physical disability level	qPCR	AFB (nerve)
PNL	male	37	2	Positive	Positive
PNL	male	72	0	Negative	Positive
PNL	male	60	1	Positive	Positive
PNL	female	41	2	Negative	Positive
PNL	female	72	0	Positive	Positive
PNL	male	36	2	Negative	Positive
PNL	female	63	1	Positive	Positive
Vasculitis	female	56	NA	Negative	Negative
Vasculitis	female	74	NA	Negative	Negative
Vasculitis	female	83	NA	Negative	Negative
Vasculitis	female	42	NA	Negative	Negative
Vasculitis	female	66	NA	Negative	Negative
Vasculitis	female	63	NA	Negative	Negative
Vasculitis	female	65	NA	Negative	Negative
Vasculitis	female	61	NA	Negative	Negative
Vasculitis	male	74	NA	Negative	Negative

(n = 16). PNL, Pure Neural Leprosy; AFB, Acid-fast bacilli; NA, Not applied.

To characterize histopathological changes, nerve samples were available for histological processing (**Supplementary Table S1**), stained with the hematoxylin and eosin, and visualized under by a light microscopy following previously published protocol (11). Samples were also subjected to qPCR and among the samples of PNL group, 4 were positive for qPCR and all samples were acid fast bacilli (AFB) positive, which was the sample selection criteria. Exclusion criteria were patients with coinfection, metabolic comorbidities such as diabetes and using corticosteroids at the time of the biopsy.

The study was approved by the Instituto Oswaldo Cruz ethics committee, CAAE number: 61142116.8.0000.5248.

### 2.2 RNA isolation

Nerve samples that had been snap-frozen in liquid nitrogen, were thawed on wet ice and homogenized using a Polytron Homogenizer PT3100 (Kinematica AG, Switzerland) in the presence of TRIzol Reagent. RNA extraction was performed according to the standard protocol provided by the manufacturer (Ambion, Thermo Fisher Scientific, MA, USA). Residual DNA contamination was removed using the DNA-free kit, following the manufacturer’s instructions (Thermo Fisher Scientific Inc., MA, USA). The integrity of the isolated RNA was evaluated using 1% agarose gel electrophoresis and the TapeStation RNA ScreenTape system (Agilent Technologies, CA, USA).

### 2.3 Library preparation and Illumina mRNA sequencing

RNA sequencing libraries were constructed using the NEBNext Ultra™ RNA Library Prep Kit (New England Biolabs, MA, USA), with ribosomal RNA depletion via the NEBNext^®^ Poly(A) mRNA Magnetic Isolation Module (New England Biolabs, MA, USA). The libraries were subsequently sequenced in paired-end mode for 150 cycles using the Illumina NovaSeq 6000 platform at NovoGene Co.

### 2.4 RNA-sequencing analysis

Raw BCL files were converted to FASTQ format using Illumina’s bcl2fastq script. Read quality was evaluated with FastQC version 0.11.8. Transcript abundance was quantified using Salmon version 1.13.0 with quasi-mapping mode, using the human transcriptome index (salmon_partial_sa_index:default) from Ensembl/RefGenie and default settings, along with the –seqBias flag. Transcript counts were aggregated into ENSEMBL gene-level counts using the R package tximport version 1.12.0 and biomaRt version 2.40.5. To verify sample labeling, expression levels of sex-chromosome-specific genes, such as *UTY* and *XIST*, were analyzed. Dataset quality was further assessed with multidimensional scaling (MDS) plots prior to inferential analysis.

Differential gene expression (DGE) analysis was conducted using DESeq2 version 1.24.0 (with fitType = “local”) following the exclusion of genes with low expression. Nominal p-values were inspected via histograms and adjusted for multiple testing using the Benjamini-Hochberg method to control the false discovery rate (FDR). Genes were designated as differentially expressed (DE) if |log_2_ fold change| ≥ 1 and FDR ≤ 0.1. For data visualization, normalized counts were transformed into log_2_ space and plotted using ggplot2 version 3.3.0. Hierarchical clustering and heatmaps were generated using pheatmap version 1.0.12 with variance-stabilized data, gene-wise z-score scaling, and either Euclidean or Ward distance measures, paired with average or complete agglomeration methods. Gene set enrichment analysis (GSEA) and overrepresentation analysis (ORA) were performed to annotate Gene Ontology (GO) terms and Reactome pathways using clusterProfiler version 3.12.0 and org.Hs.eg.db version 3.8.2. Enrichment p-values were adjusted for multiple testing using the Benjamini-Hochberg method, with an FDR cutoff of 0.1. Principal component analysis (PCA) was performed using the top 500 most variable genes after normalization, with variance stabilization applied via DESeq2 (blinded to sample groups).

### 2.5 Immunohistochemistry

Frozen peripheral nerve fragments were cut on a Leica LM 1850UV cryostat (Leica, Wetzlar, Germany). The 5 µmµm thick sections were fixed in cold acetone and hydrated in 0.01 M phosphate buffer saline (PBS). Endogenous peroxidase was blocked in 0.3% hydrogen peroxide solution diluted in 0.01 M PBS and then washed in 0.01 M PBS. Unspecific binding sites were blocked with 0.01 M PBS solution containing 10% normal goat serum (NGS) and 0.1% bovine serum albumin (BSA). The following primary antibodies were diluted in 0.01 M PBS solution containing 1% NGS and incubated overnight at 4°C in humid chamber: anti-CD68 (1:100 Dako M0814), anti-S100 (1:100 Dako Z0311), anti-ASC (1:50 Cell Signaling Technology #13833), anti-IL1β (1:25 Cell Signaling Technology #12242) and anti- NLRP1 (1:50 Abcam; ab36852). Next, sections were washed with 0.01 M PBS and incubated in a HiDef signal amplifier solution for 20 minutes, and then washed in 0.01 M PBS and incubated in a HiDef HRP polymer detection solution (kit HiDef detection HRP polymer system, CellMarque, 954-D) for 20 minutes. Sections were washed again with 0.01 M PBS. Immunostainings were developed in 3-amino-9-ethylcarbazole solution (AEC substrate Kit, Vector Labs SK-4200). The cell nuclei were stained with Harris’ Hematoxylin. Sections were mounted with coverslips using an aqueous mounting medium (Abcam128982) and the results were analyzed under Nikon Eclipse E400 optical microscope with a plan-apochromat 40X objective (Nikon Instruments Inc., New York, USA).

## 3 Results

### 3.1 Differential gene expression and functional enrichment analysis in pure neural leprosy nerve fragments

**Figure 1:**
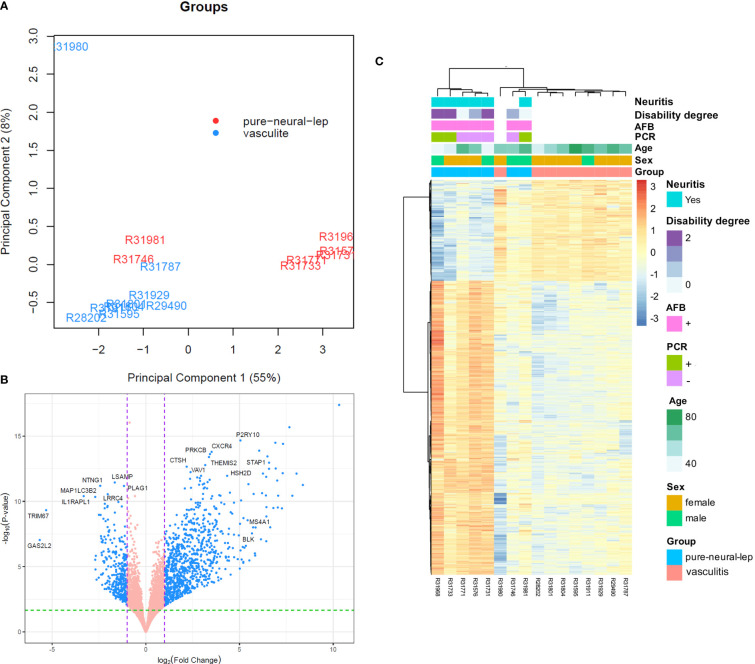
Nerve fragments from PNL patients display a distinct transcriptomic profile to that of non-leprosy controls. **(A)** Principal component analysis (PCA) of the 500 most variable genes in PNL patients (PNL; red, n=6) compared to those that were non-leprosy controls (Vasculitis; blue, n=9). **(B)** Volcano plot analysis of differentially expressed genes in PNL vs. non-Leprosy controls. Differentially expressed genes are depicted in blue. **(C)** Heatmap showing the differentially expressed genes (DEG) in the nerve fragments from PNL (pure-neural-lep) patients over non-leprosy controls (vasculitis). In addition to comparing patients with PNL to the control group (vasculitis), the correlation of gene expression with the presence of neuritis, disability degree, AFB, qPCR, age and sex is presented, showing that gene expression is not influenced by these variables. Expression levels are represented by a scale of log_2_FC from -3 (dark blue) to 3 (bright red). AFB, acid fast bacilli.

Genome-wide transcriptional profiles were generated from nerve fragments obtained from individuals in the PNL group (n = 7) and non-leprosy controls (n = 9). A dataset of 15,165 expressed genes was compiled, and PCA of the 500 most differentially expressed genes successfully separated the samples into two distinct groups. However, two PNL samples were positioned between these two groups along the first two principal components (**Figure 1A**). In total, 1,199 genes were identified as differentially expressed (DE) in PNL nerve fragments compared to controls, with 334 genes downregulated and 568 upregulated (|log_2_ fold change| ≥ 1, FDR ≤ 0.1) (**Supplementary Table S2**). Among the downregulated genes, two showed more than a 32-fold reduction in expression in PNL patients: *GAS2L2* (log_2_FC = -5.66, adj. p = 0.001), involved in cytoskeletal regulation, cell cycle, apoptosis, and differentiation; and *TRIM67* (log_2_FC = -5.32, adj. p = 0.001), a regulator of autophagy, apoptosis, and innate immune responses. Additionally, *IL1RAPL1* (log_2_FC = -3.31, adj. p < 0.001), a member of the interleukin-1 receptor family associated with synaptopathy, showed an eightfold reduction (14–16). Other notable downregulated genes included *MAP1LC3B2* (log_2_FC = -2.7, adj. p < 0.001), related to autophagy; *NTNG1* (log_2_FC = -2.44, adj. p = 0.001), involved in somatosensory neuron function; and *LRRC4* (log_2_FC = -2.0, adj. p = 0.001), which regulates excitatory synapse formation and axon differentiation (**Figure 1B**) (17). Conversely, the top 100 upregulated genes were predominantly immunoglobulin-related (**Supplementary Table S2**). A heatmap of all 1,041 DEGs was generated, highlighting these patterns (**Figure 1C**).

**Figure 2:**
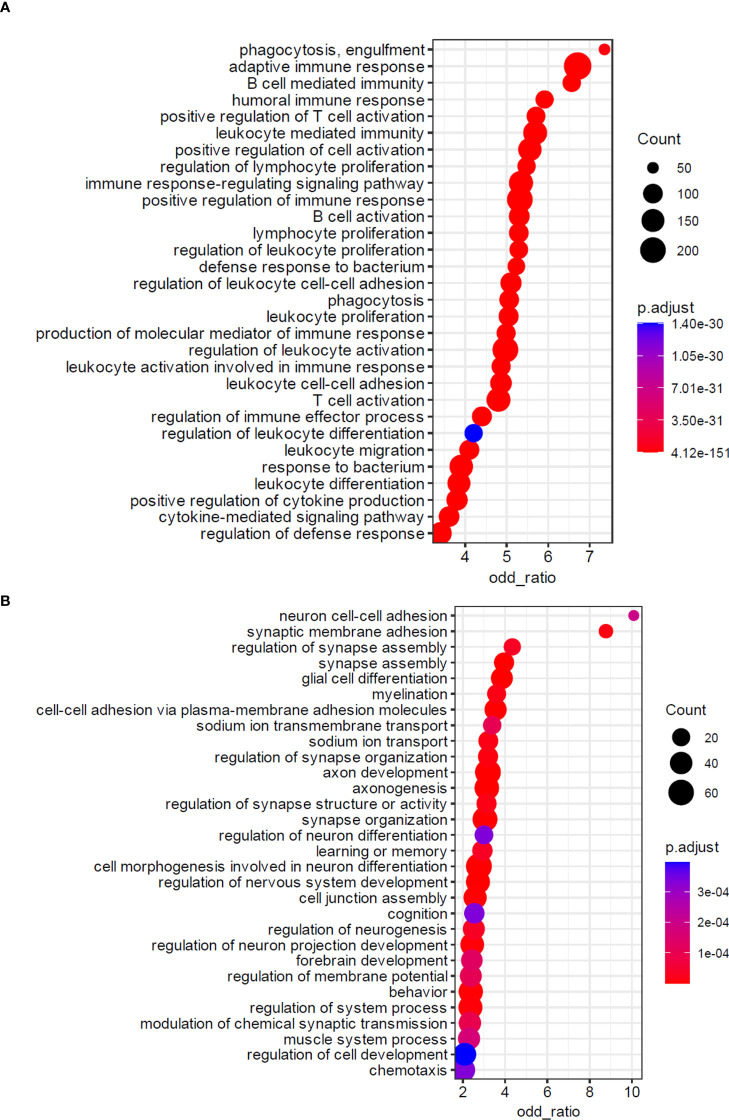
Biological processes enriched in the nerve fragments transcriptomics of patients experiencing PNL. Dotplot of enriched biological processes associated with **(A)** positively and **(B)** negatively regulated genes in PNL patients obtained by the overrepresentation analysis (ORA). Pathways are depicted in a hierarchy according to the enrichment ratio. Circle size (Count) and color indicate, respectively, the number of genes associated with the pathway and the adjusted P-value. The square color indicates the p.adjust value according to the scale.

Functional analysis of the differentially expressed genes in nerve fragments from the PNL group compared to non-leprosy controls revealed distinct biological enrichments. In the PNL group, upregulated genes were predominantly associated with the positive regulation of adaptive immune responses, including numerous immunoglobulin-related genes. Additional enriched pathways included processes such as leukocyte activation, leukocyte-mediated immunity, leukocyte differentiation, and responses to bacterial infections (**Figure 2A**). In contrast, nerve fragments from non-leprosy controls exhibited significant enrichment of genes related to neural development and organization. These included genes involved in axon development, axonogenesis, synapse organization, cell morphogenesis during neuronal differentiation, and peripheral nervous system development (**Figure 2B**). These findings further corroborate the pathological changes in host cells caused by *Mycobacterium leprae*, which are linked to the degenerative alterations observed in the nerves (**Supplementary Figure 1**).

#### 3.1.1 A deficiency in the expression of genetic genes essential for autophagy may increase the inflammasome pathways in the nerves of PNL patients

**Figure 3:**
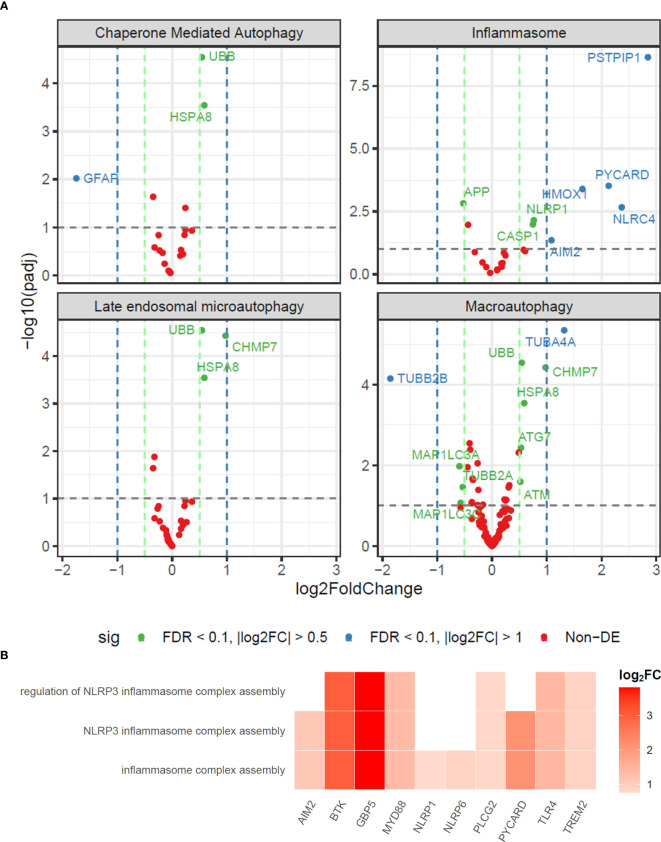
Nerve fragments from PNL patients display a deficiency in the expression of autophagy genes and an increase in the inflammasome pathways. **(A)** Volcano plot analysis of differentially expressed genes in inflammasome and autophagy pathways by PNL vs. non-Leprosy controls. Differentially expressed genes are depicted in blue (FDR < 0,1 |log_2_FC| > 1) and green (FDR < 0.1 |log_2_FC| > 0.5). **(B)** Heatplot of enriched biological processes associated with positively regulated genes in the PNL patients obtained by over-representation analysis (ORA). Values of log_2_FC are color-graded according to the scale.

Deficiencies in autophagy-related proteins induce the aberrant activation of inflammasomes, causing severe tissue damage (18). We showed that while macroautophagy genes like *MAP1LC3B2* were downregulated, inflammasomes-related genes like *NLRC4* (log_2_FC = 2.37, adj. p = 0.002), *PYCARD* (log_2_FC = 2.13, adj. p = 0.0003), *AIM2* (log_2_FC = 1.08 adj. p = 0.04) and *PSTPIP1* (log_2_FC = 2.85, adj. p < 0.001) were upregulated in nerve fragments from PNL group *vs.* non-leprosy controls (**Figure 3A**). Furthermore, functional analysis of the genes significantly enriched in nerve fragments from PNL group by ORA showed genes involved in biological processes like “inflammasome complex assembly”, “NLRP3 inflammasome complex assembly” and “regulation of NLRP3 inflammasome complex assembly”. These genes were *AIM2, BTK, GBP5, MYD88, NLRP1, NLRP6, PLCG2, PYCARD, TLR4* and *TREM2* (**Figure 3B**). During the clinical course of leprosy, inflammatory episodes may occur and may be associated with an improvement of neural damage. Our previous data demonstrated that during type 1 reaction in multibacillary patients, an increased expression of inflammasome proteins was correlated with autophagy impairment (19), which suggests that the autophagy-inflammasome regulation could be associated with the immunopathogenesis of nerve damage in leprosy.

### 3.2 The inflammasome components expression is associated with PNL

**Figure 4:**
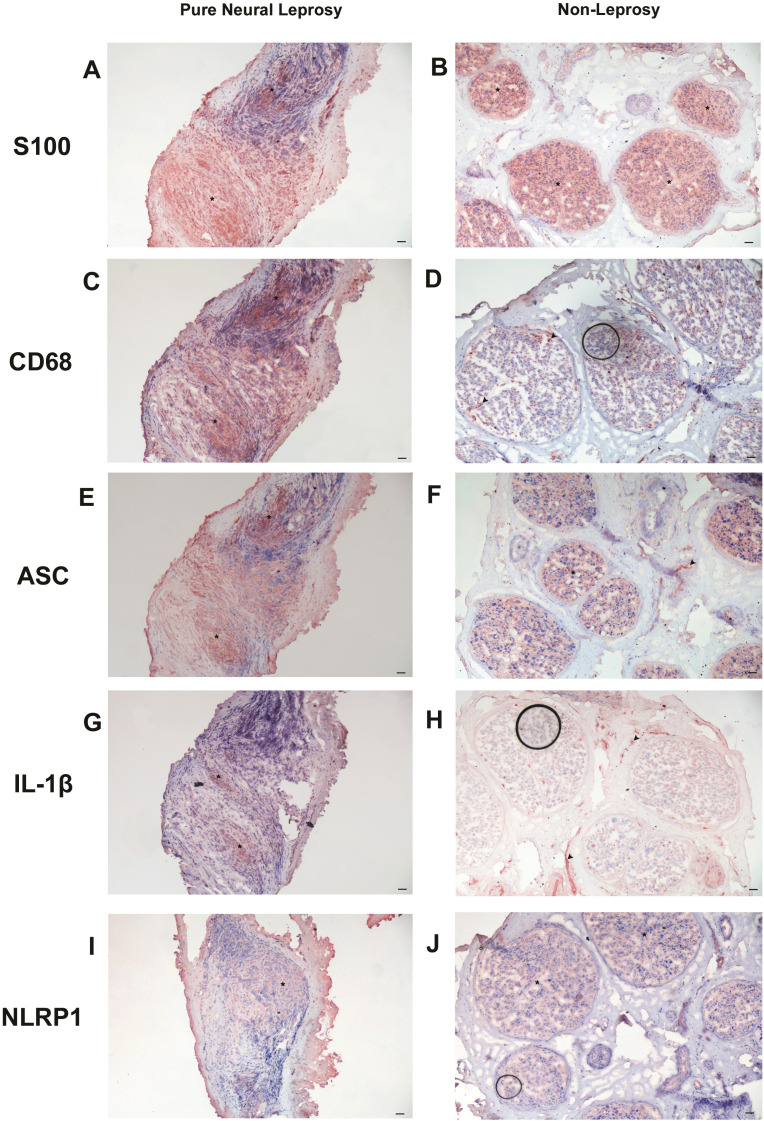
The inflammasome components are increased in nerve fragments from PNL. **(A, B)** Expression of S100; **(C, D)** CD68; **(E, F)** ASC; **(G, H)** IL-1β; and **(I, J)** NLRP-1 was detected by immunohistochemistry. Epineurium (asterisk); Vessels (arrows). Representative images are shown. Bars: 50 µm.

Inflammatory infiltration in perineurium and endoneurium compartments, fiber loss, fibrosis, and/or epithelioid granuloma formation are some of the most common histopathologic alterations observed in neural leprosy (**Supplementary Figure 2**). We next aimed to validate the inflammasome components in nerve fragments from PNL and non-leprosy control. The immunohistochemical data presented in **Figure 4** shows that samples from PNL nerves had a loss of nerve parenchyma architecture and an intense inflammatory infiltrate distributed in the endoneurium, observed by intense CD68 marking (macrophage marker) and decreased S100 (Schwann cell marker) in the endoneurium compared to non-leprosy control (**Figures 4A–D**). ASC, an inflammasome adapter protein, was most strongly expressed in the endoneurium of the PNL nerves (**Figure 4E**), whereas in the non-leprosy nerves, the labeling was localized in the epineurium vessels, probably due to the vasculitis neuropathy (**Figure 4F**). In addition to ASC, the expression of IL-1β, a major product of the inflammasome, followed the same labeling pattern. NLRP1, on the other hand, marked only endoneurial cells of PNL nerves (**Figures 4G–J**).

## 4 Discussion

Leprosy is a complex and multifactorial disease caused by different triggers, affecting Schwann cells, the glial cells of the PNS, and other non-neuronal cells in the neural microenvironment (e.g. macrophages). Since this is a condition unique to leprosy, knowledge of the mechanisms that trigger nerve damage remains poorly understood (20, 21). Schwann cells are believed to be directly involved in the pathogenesis of nerve damage in leprosy, however, it is not known to what extent the inflammatory microenvironment within and around nerves may influence nerve damage (22).

Understanding the events that occur in the nerves of patients with leprosy neuropathy is essential for identifying markers that can be used to evaluate diagnostic strategies or that can function as targets for the development of new therapeutic alternatives, which can contribute to the control of leprosy neural damage. Currently, the infection of Schwann cells by *M. leprae* has been associated with the subversion of the host cell’s energy metabolism to the detriment of the bacillus’s survival like alterations in the glucose/lactate metabolic pathway (23, 24), lipid/cholesterol accumulation (25, 26), mitochondrial dysfunction (27), and myelin dismantling (28). Some of these changes have also been confirmed in leprosy patients and are believed to contribute to the ongoing neuropathy, tissue fibrosis, and loss of nerve function observed in individuals with leprosy (29–32). Although these events may stem from disruptions in the Schwann cells’ supportive role within the nerve, the associated changes in the gene expression profile remain largely unknown. Recently, our group showed by RT-qPCR a molecular signature associated with neural damage in early stages of PNL patients (13). However, it is necessary to focus on better understanding the PNL, since it is yet another bottleneck in clinical diagnosis and therapeutic intervention, increasing the risk of sequelae and maintaining continued transmission of the bacillus. Its clinical diagnosis is challenging since patients share characteristics with other common pathologies that must be taken into account for differential diagnosis (9, 10).

Here we applied a transcriptomic approach to identify the molecular signature for the discrimination of patients with leprosy neuropathy from patients with other peripheral neuropathies. Our RNA-seq data showed a lower expression of genes important for good maintenance of neuronal architecture like *GAS2l2*, *TRIM67*, *MAP1LC3B2*, *NTNG1*, *LRRC4* and *IL1RAPL1*. The least enriched genes in PNL patients are involved in the modulation and dynamics of the cytoskeleton, axonogenesis and axonal development, neuronal differentiation, synapses and development of the peripheral neuronal system (**Figure 2B**). This is consistent with the pathogenesis of leprosy neuropathy, since the invasion and slow multiplication of *M. leprae* trigger a subacute and later chronic inflammatory response that culminates in a destructive effect with loss of nerve fiber integrity (33). In previous studies, our group demonstrated through immunostaining in biopsies of patients with PNL the reduced expression of neural markers and loss of myelinated fibers. Immunoperoxidase staining against neural markers of axons, neurofilament, nerve growth factor receptor (NGFr), protein 9.5 gene product and Schwann cells (myelin basic protein, S-100 protein and NGFr) was reduced (34). Furthermore, *M. leprae* stimulates Schwann cells to secrete matrix metalloproteinases (MMP) preceding inflammatory infiltration, suggesting that the imbalance of matrix MMP-2 and MMP-9 also contributes to the final configuration of leprosy neuropathy (31, 35). Moreover, recent investigations have shown a descriptive *in situ* quantitative morphological overview of the three concomitant mechanisms involved in leprosy nerve damage, specifically axonal atrophy, Wallerian degeneration and demyelination (36).

In addition to being involved in neurogenesis, some of these lower expression genes are closely related to the autophagic process, a degradation pathway with essential roles in cellular homeostasis and tissue survival, including nervous tissue (37–39). It is now known that macroautophagy is essential for the removal of myelin in the early stages of nerve injury by a mechanism called myelinophagy. In the cytoplasm of Schwann cells, myelin debris resulting from the demyelination process is organized into ovoids that undergo lysosomal degradation via autophagy (40, 41). However, the metabolic implication of myelinophagy for the physiology of *M. leprae* was ignored until recently. A study demonstrated that *M. leprae* benefits from the myelinophagy process for its survival within Schwann cells. Demyelination caused by the interaction of *M. leprae* with primary myelinating mouse Schwann cells (mSCs) accelerated the myelinophagy process and lipid derivatives favored the survival of the bacillus (28).

On the other hand, autophagy plays an important response induced by intracellular pathogens (xenophagy), it contributes to the systemic immune response by modulating inflammation, cytokine production, as well as adaptive immunity (27). Genetic alterations in components of the autophagy pathway result in autoinflammatory and neurodegenerative disorders (42). Recently (43), demonstrated that patients with deficiency in the expression of the *MAP1LC3B2* gene, an autophagy-associated inborn error of immunity, when infected with the varicella-zoster virus (VZV), presented a reduction in the activation of autophagy pathway, resulting in increased viral replication and cell death in peripheral blood cells. This same effect was observed in *in vitro* experiments, using human neuroblastoma cell line (SH-SY5Y) infected with VZV, which highlights the neuroprotective role of autophagy.

Corroborating our studies, Zhang et al. (2021) by using a murine model of encephalomyelitis (EAE), identified that the proteins encoded by *LRRC4* gene have a neuroprotective role. Loss of this protein triggers worsening demyelination in the spinal cord and accelerates the recruitment of inflammatory cells, such as lymphocytes, exacerbating the injury. This mechanism is mediated by increased NF-kB, since LRRC4 is an important repressor of this transcription factor. Thus, deficiency in LRRC4 expression stimulates the increase in pro-inflammatory cytokines via NF-kB (44).

On the other hand, autophagy and inflammasomes are functionally interconnected. Autophagy may negatively regulate inflammation by degrading inflammasome components by autophagosomes (18). We showed that while macroautophagy genes like *MAP1LC3B2* being downregulated, inflammasomes-related genes like *AIM2*, *NLRC4*, *NLRP1 PYCARD* and *CASP1* were differentially expressed in nerve fragments from PNL group (**Figure 3A**). Moreover, ORA analysis showed inflammasome genes involved in enriched biological processes associated with positively regulated genes in the PNL patients (**Figure 3B**).

Inhibition of autophagy has also been shown to strongly activate the inflammasome, as the absence of autophagy leads to the accumulation of endogenous stimuli (a second signal) necessary for inflammasome activation (45). By the way, our immunohistochemical data in nerve fragments corroborated the transcriptomic analysis, showing an increased inflammatory infiltrate, mainly in the endoneurium, through macrophage markers (CD68), as well as inflammasome proteins, such as ASC, IL-1β and NLRP-1 in PNL patients compared to non-leprosy control (**Figure 4**). This finding corroborates a previous study by our group, which showed that autophagy can contribute to the control of bacillary load in leprosy patients (46) and that blocking autophagy in skin lesion cells from multibacillary patients is associated with the establishment of reactional episodes by a mechanism associated with the induction of inflammasome (19). It is worth highlighting that leprosy reaction episodes are responsible for worsening neural impairment (47). Furthermore, an increase in serum levels of IL-1β was demonstrated in patients with leprosy neuropathy (48). Here we show for the first time the relationship of inflammasomes with leprosy neuropathy. Our data suggest that *M. leprae* may influence the expression of autophagy genes, important for the regulation of inflammasome proteins and consequently favor the activation of this platform in peripheral nerves and, therefore, may be involved in the immunopathogenesis of leprosy neuropathy.

Furthermore, *PSTPIP1*, a gene involved in autoinflammatory disease (49), was one of the most upregulated genes in the analysis of differentially expressed genes in inflammasome pathways by PNL patients (**Figure 3A**). Besides, *PSTPIP1* is in the 35th position globally of the most upregulated genes (**Supplementary Table S2**). It is a gene that encodes a regulatory protein phosphatase that modulates the activation of T cells and phagocytes, as well as the organization of the cytoskeleton (50). In addition, this protein is involved in the overactivation of the innate immune system with intense production of IL-1β and IL-18, via inflammasome activation, as well as neutrophilic infiltration (51). Alterations in this gene have been associated with the occurrence of Sterile Pyogenic Arthritis (PAPA Syndrome), Pyoderma Gangrenosum, Acne and Behçet’s Syndrome (50).

Together these data suggest that *M. leprae* may influence the expression of autophagy genes, important for the regulation of inflammasome proteins and consequently favor the activation of this platform in peripheral nerves and, therefore, may be involved in the immunopathogenesis of leprosy neuropathy.

The immune response provoked in the nervous microenvironment against bacilli has been widely studied as the main key component that can lead to distinct clinical manifestations (6). However, peripheral nerve inflammation does not necessarily occur due to direct infection of Schwann cells or other nerve cells by the pathogen, but also due to its indirect effects. These effects include the formation of anti-pathogen antibodies forming immune complexes, which cross from the systemic circulation to the nerve resulting in demyelination and axonal injury by complement pathway activation (52, 53). These antibodies in affected tissues lead to disabilities and impact the quality of life of leprosy patients. Interestingly, our data showed a highly inflammatory profile involved with immunoglobulins, adaptive immune response and leukocyte activation genes in nerve biopsies from PNL patients compared to respective control groups (**Figure 2A**). Moreover, analysis of differentially expressed genes in PNL *vs.* non-leprosy controls showed immunoglobulins genes among the top 100 of the upregulated genes, in addition to the expression of complement components, corroborating the effects described above (**Figure 1B**, **Supplementary Table S2**). These data suggest the involvement of the humoral immune response in the pathogenesis of leprosy-related neural damage. Future studies are needed to better elucidate the role of this response in the establishment of neuropathy.

This study provides valuable insights into the molecular pathogenesis of PNL, highlighting the relevance of inflammasome activation and autophagy dysfunction in the nerve damage associated with leprosy neuropathy. However, a key limitation of our study is the cohort size, as a larger sample could enhance statistical power and allow for greater generalization of the results. The number of samples analyzed, however, is proportional to the prevalence of the diseases investigated, which are considered rare. This makes obtaining a larger number of clinical specimens particularly challenging, especially given that nerve biopsy is an invasive procedure not routinely performed in clinical practice. Consequently, this limitation restricts the inclusion of additional peripheral neuropathies in the study. Nevertheless, investigating different peripheral neuropathies could offer a broader perspective on inflammatory patterns. Notably, the vasculitis group consisted predominantly of female patients, whereas the PNL group was mostly male. Despite these differences, the heatmap (**Figure 1C**) showing the DEGs indicates that sex and age did not alter the gene expression pattern within or between the groups.

The qPCR technique has contributed to the diagnosis of cases in which the clinical and histopathological data were not conclusive evidence of PNL (10). In the present investigation, three of the seven PNL patients showed the presence of AFB with a negative qPCR (**Table 1**). Since nerve biopsy is an invasive procedure, the collected tissue must be used efficiently to maximize diagnostic yield. Therefore, the biopsied nerve fragment is divided for both histopathological and molecular analysis. As histopathology remains the gold standard for diagnosing PNL in this context, most of the sample is typically allocated for this purpose. Additionally, due to the low bacillary load in paucibacillary individuals, the fragment sent for qPCR analysis may not always contain bacilli, potentially leading to false-negative results. Thus, in addition to detection by qPCR and AFB, a combination of histopathological findings, clinical evaluation and electroneuromyography studies contribute to the diagnosis of PNL.

The present study provides new information associated with the genes and/or pathways modulated by *M. leprae* in peripheral nerves of leprosy patients. The identification of this genetic profile may contribute to the better understanding of the pathogenesis of leprosy neuropathy with the aim of identifying these pathways as targets for the development of effective therapeutic strategies to improve differential diagnosis and pharmacological targets to treatment.

## Data Availability

The datasets presented in this study can be found in online repositories. The names of the repository/repositories and accession number(s) can be found below: GSE287973 (GEO)”- not publicly available.

## Supplementary material

Supplementary Figure 1 Gene Ontology Biological Processes associated with DEG by GSEA. Gene set enrichment analysis (GSEA) highlighting the main biological processes from Gene Ontology enriched in DEG between PNL patients vs. non-leprosy controls. Circle size (Count) and color indicate, respectively, the number of genes associated with the pathway and the adjusted P-value. The square color indicates the p.adjust value according to the scale. NES, normalized enrichment score.

Supplementary Figure 2 Histopathologic alterations observed from PNL and non-leprosy patients. Nerve fragments were collected from PNL and non-leprosy patients and the Hematoxylin-Eosin (HE) routine was performed to characterize the architecture of the nerve parenchyma. Cross section from PNL showing **(A)** fibrosis (yellow star), thickening of the perineurium (yellow arrow) and mononuclear inflammatory infiltrate in epineurium (red circle). Mononuclear inflammatory cells are also present in epineurium and endoneurium (100x magnified); **(B)** intense mononuclear inflammatory infiltrate in endoneurium (red circle) and endoneurial fibrosis (yellow star) (40x magnified) and **(C)** marked mononuclear inflammatory infiltrate around vessels, fascicles and in fibroadipose tissue. The inflammatory infiltrate is also present in the perineurium and endoneurium, which exhibit outlines of granulomas (100x magnified). In contrast, cross section from non-leprosy showing **(D)** a mild to moderate mononuclear inflammatory infiltrate (red arrows) around vessels (red circles) of the epineurium (200x magnified); **(E)** a mild mononuclear inflammatory infiltrate with accentuation (red arrows) around the vessels (red circles), mainly of the epineurium (100x magnified) and **(F)** some foci of mononuclear inflammatory infiltrate around vessels (black circles) and thickening of the perineurium (yellow arrows) (100x magnified). Representative images are shown.
