# Supplementary figures and images for: Gene expression profiling in pure neural leprosy: insights into pathogenesis and diagnostic biomarkers
Source: Front Immunol. 2025 May 12;16:1550687. doi: 10.3389/fimmu.2025.1550687 (PMC12104059; doi:10.3389/fimmu.2025.1550687)

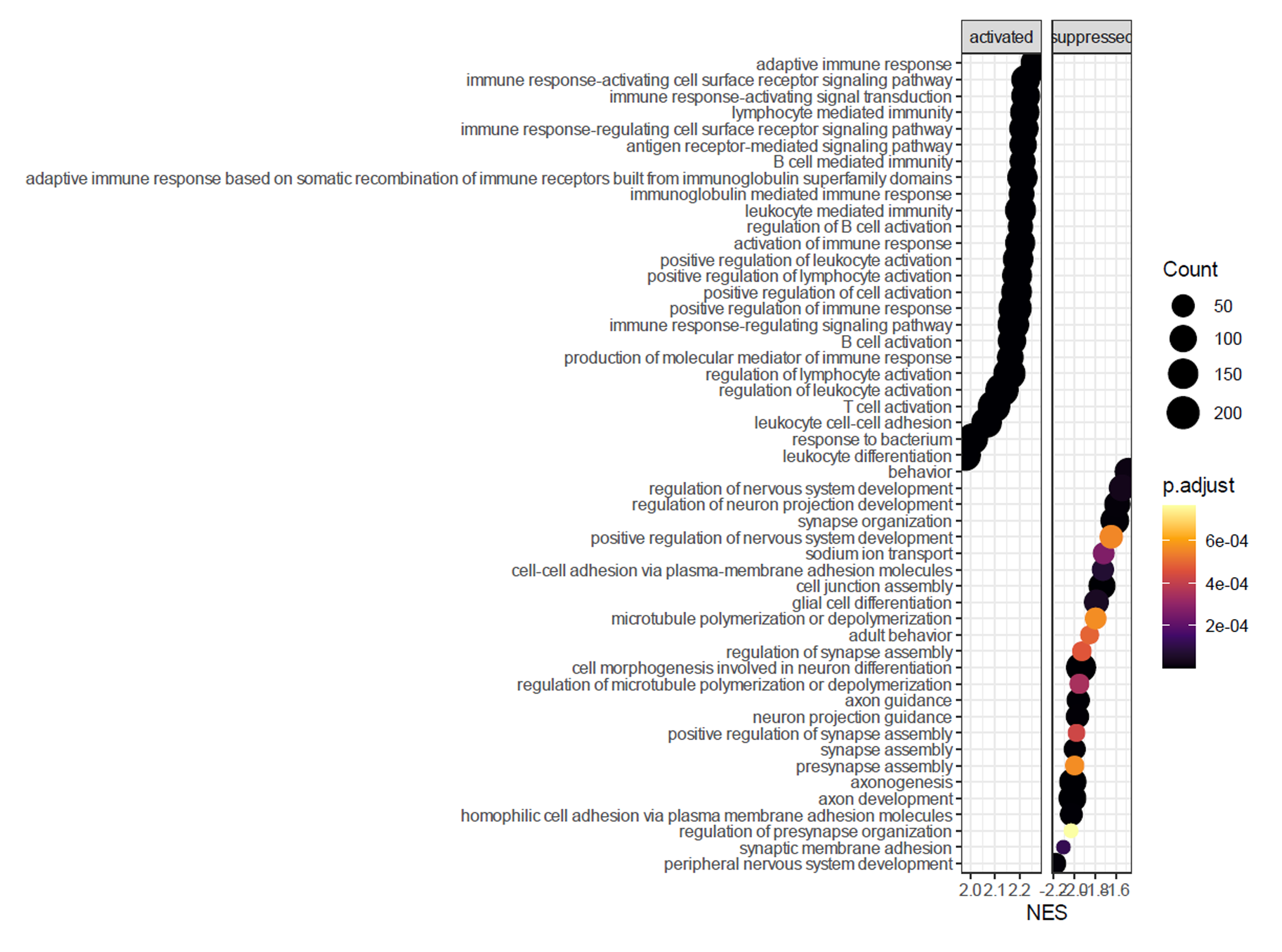

Supplement: Supplementary Figure 1 — Gene Ontology Biological Processes associated with DEG by GSEA. Gene set enrichment analysis (GSEA) highlighting the main biological processes from Gene Ontology enriched in DEG between PNL patients vs. non-leprosy controls. Circle size (Count) and color indicate, respectively, the number of genes associated with the pathway and the adjusted P-value. The square color indicates the p.adjust value according to the scale. NES, normalized enrichment score. [file Image1.tif]

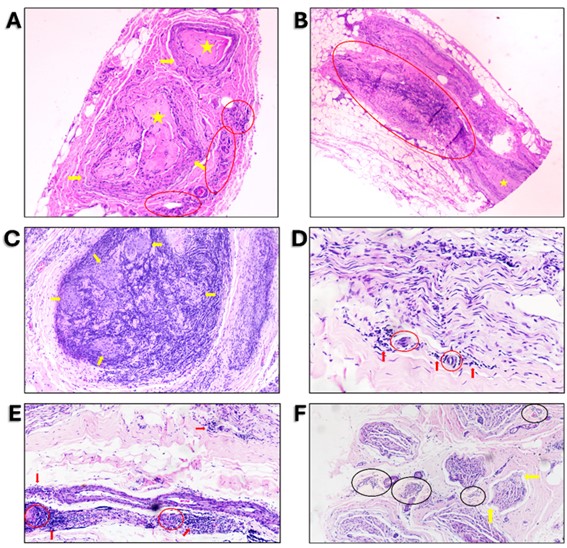

Supplement: Supplementary Figure 2 — Histopathologic alterations observed from PNL and non-leprosy patients. Nerve fragments were collected from PNL and non-leprosy patients and the Hematoxylin-Eosin (HE) routine was performed to characterize the architecture of the nerve parenchyma. Cross section from PNL showing (A) fibrosis (yellow star), thickening of the perineurium (yellow arrow) and mononuclear inflammatory infiltrate in epineurium (red circle). Mononuclear inflammatory cells are also present in epineurium and endoneurium (100x magnified); (B) intense mononuclear inflammatory infiltrate in endoneurium (red circle) and endoneurial fibrosis (yellow star) (40x magnified) and (C) marked mononuclear inflammatory infiltrate around vessels, fascicles and in fibroadipose tissue. The inflammatory infiltrate is also present in the perineurium and endoneurium, which exhibit outlines of granulomas (100x magnified). In contrast, cross section from non-leprosy showing (D) a mild to moderate mononuclear inflammatory infiltrate (red arrows) around vessels (red circles) of the epineurium (200x magnified); (E) a mild mononuclear inflammatory infiltrate with accentuation (red arrows) around the vessels (red circles), mainly of the epineurium (100x magnified) and (F) some foci of mononuclear inflammatory infiltrate around vessels (black circles) and thickening of the perineurium (yellow arrows) (100x magnified). Representative images are shown. [file Image2.jpeg]
